# Ti_3_C_2_T_*x*_ 2D and 0D MXene Cocatalysts on CuO for Enhanced Photocatalytic Hydrogen Evolution

**DOI:** 10.1021/acs.energyfuels.5c01244

**Published:** 2025-06-06

**Authors:** Lu Chen, Taotao Qiang, Matyas Daboczi, Yasmine Baghdadi, Salvador Eslava

**Affiliations:** 1 Institute of Biomass & Functional Materials, Shaanxi University of Science & Technology, Xi’an, Shaanxi 710021, P.R. China; 2 Department of Chemical Engineering, 4615 Imperial College London, London SW7 2AZ, United Kingdom

## Abstract

Cocatalysts play a crucial role in photocatalytic reactions, and titanium carbide MXene (Ti_3_C_2_T_*x*_) is a promising alternative to expensive noble metal cocatalysts. Herein, we coupled a copper­(II) oxide (CuO) semiconductor with Ti_3_C_2_T_*x*_ in two dimensions, nanosheets (T2D) and quantum dots (T0D), forming T2D/CuO and T0D/CuO composite photocatalysts. The effects of size, morphology, and energetics of the different Ti_3_C_2_T_*x*_ forms were investigated in relation to their photocatalytic hydrogen production rates. The T0D/CuO sample achieved a hydrogen production rate of 2174 (±189) μmol g^–1^ h^–1^, which is 19 and over 100 times higher than those of T2D/CuO samples and pure CuO, respectively. The enhanced performance of T0D/CuO compared to T2D/CuO can be attributed to a smaller particle size, improved light absorption, larger specific surface area, and a deeper T0D work function promoting charge separation for photocatalytic reactions. These results highlight the impact of the different dimensionalities of titanium carbide MXenes on the photocatalytic performance of composites and point to promising avenues to achieve efficient photocatalytic systems.

## Graphical abstract

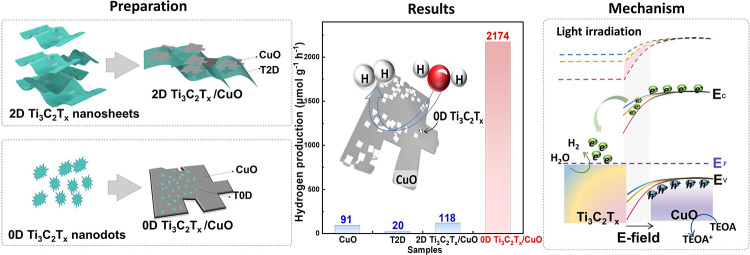

## 1 Introduction

Photocatalysis is a green technology for converting solar into chemical energy and has been successfully used in water pollution treatment,^1^ seawater desalination,^2^ and antibacterial^3^ and energy applications.^4^ However, further research is required to exploit the full potential of photocatalysis. One challenge in this research area is the fast recombination of photogenerated electrons (e^–^) and holes (h^+^), which largely hinders the relatively slow (ms to s timescale) photocatalytic processes, such as water splitting.^5^ Introducing cocatalysts is a promising method to suppress the e^–^/h^+^ recombination in photocatalysts. Cocatalysts often serve as electron acceptors, facilitating the effective separation of carriers owing to their excellent conductivity.^6^ Simultaneously, cocatalysts can also mediate in certain catalytic paths and boost their kinetics. For instance, Wu’s work^7^ showed that cocatalysts can assist in capturing H^+^ and H_2_O generated during the hydrogen oxidation reaction, thereby accelerating interfacial catalytic kinetics. Platinum (Pt) is a commonly used cocatalyst that boosts the utilization of photoinduced electrons for the reduction of H^+^ to H_2_ gas.^8^ Nevertheless, Pt is a scarce, expensive, noble metal; therefore, new materials need to be explored.^9^ To achieve high photocatalytic activity, cocatalysts need to avoid parasitic light absorption while ensuring favorable interfacial energetics that boost charge separation and utilization in the catalytic process of interest.^10–14^

MXenes are novel 2D transitional metal carbides, nitrides, or carbonitrides with a great potential for use as inexpensive cocatalysts in photocatalytic systems.^15^ Their general formula is M_*n*+1_X_*n*_T_*x*_, where M represents a IIB-VIB metal (Ti, Sc, V, or Mo), X is a C or N atom, and T_*x*_ represents surface functional groups (−F, −OH, or −O). MXenes are prepared by etching the A layer of a MAX phase with the chemical formula M_*n*+1_AX_*n*_, where A is Al, P, Si, S, or Ga. Titanium carbide MXene (Ti_3_C_2_T_*x*_) was the first reported MXene and has been applied in different scientific fields, such as lithium-ion batteries,^16^ supercapacitors,^17^ electrocatalysis,^18^ electromagnetic shielding,^19^ and biomedicine.^14^ Ti_3_C_2_T_*x*_ has also been used as a cocatalyst in photocatalysts for hydrogen evolution and CO_2_ reduction.^20^ Multilayer Ti_3_C_2_T_*x*_ (T3D) is often used as a substrate for composites,^21^ while monolayer Ti_3_C_2_T_*x*_ (T2D) can offer Ti vacancies, thereby enhancing its nucleophilic properties or improving reactivity.^22,23^

The majority of Ti_3_C_2_T_*x*_-based composites use T3D or T2D nanosheets as substrates for the growth of composite materials.^24–28^ Zero-dimensional Ti_3_C_2_T_*x*_ quantum dots (T0D) derived from MXenes have also attracted research interest.^29,30^ T0D possesses hydrophilicity, conductivity, and biocompatibility, while exhibiting optical tunability resulting from size tunability, which can be used for sensors and light-emitting devices by controlling the absorption and emission spectra.^31–34^ T0D also has the potential to be used as cocatalysts for the formation of photocatalytic composites.^35^

Compared to T3D and T2D, T0D has not been thoroughly investigated as a photocatalytic cocatalyst.^36–38^ Current studies indicate that its photocatalytic performance is closely linked to surface defects, which provide additional active sites for carrier separation, and its electronic structure,^39,40^ which influences energy band position and catalytic kinetics. However, the effect of Ti_3_C_2_T_*x*_ morphology on its cocatalytic ability has received less attention. Additionally, as a common semiconductor, CuO is widely used in gas sensors due to its sensitivity to gases and its performance in photocatalytic hydrogen production, and the potential synergistic effects with Ti_3_C_2_T_*x*_ remain to be thoroughly examined.

In this work, we present photocatalytic composites of T2D and T0D with CuO (T2D/CuO and T0D/CuO) and compare their morphology, optoelectronic properties, and related photocatalytic hydrogen production performance. A wide range of characterization techniques are used, such as X-ray photoelectron spectroscopy (XPS), transmission electron microscopy (TEM), and nitrogen sorption analysis for their composition, morphology, and surface area properties. Further insights into the photocatalytic mechanism are investigated through ambient photoemission spectroscopy and Kelvin probe measurements. Owing to the reduced dimensions of 0D Ti_3_C_2_T_*x*_, T0D/CuO outperforms T2D/CuO by 19 times in photocatalytic hydrogen production, achieving a remarkable hydrogen production rate of 2174 (±189) μmol g^–1^ h^–1^. The scientific findings in this work will facilitate the design and development of Ti_3_C_2_T_*x*_-based photocatalytic composites.

## 2 Experimental Section

### 2.1 Chemicals and Materials

Titanium aluminum carbide (Ti_3_AlC_2_, 98.0%) was obtained from Macklin Biochemical Technology in Shanghai, China. Lithium fluoride (LiF, AR), hydrochloric acid (HCl, 12 M), and sodium hydroxide (NaOH, AR) were obtained from Zhanyun Shanghai Chemical Co., Ltd. Copper nitrate (Cu­(NO_3_)_2_·3H_2_O, AR) was purchased from Damao Tianjin Chemical Co., Ltd. Ammonia solution (NH_3_·3H_2_O, 25%) was purchased from Tianli Tianjin Chemical Co., Ltd. All solvents and chemicals were of analytical grade and used as received without further purification.

### 2.2 Preparation of Multilayered Ti_3_C_2_T_*x*_ (T3D)

Multilayer Ti_3_C_2_T_*x*_ (T3D) was obtained by acid etching and ultrasound delamination. Two g of LiF was added to a 100 mL Teflon-lined vessel containing 50 mL of 9 M HCl, and the mixture was vigorously stirred for 30 min. Then, 2 g of Ti_3_AlC_2_ was added slowly to the above solution for acid etching of the aluminum layers at 35 °C for 24 h under stirring. After etching, the precipitate was centrifuged and washed twice with 160 mL of 1 M HCl to remove F^–^ anions and then washed with deionized water 6–8 times until the pH became higher than 6. The precipitate was dried at 60 °C for 12 h in a vacuum oven to obtain T3D.

### 2.3 Preparation of Monolayer Ti_3_C_2_T_*x*_ (T2D)

One g of the prepared T3D was dispersed in 50 mL of deionized water, kept under nitrogen gas, sonicated in an ice–water bath for 90 min, and finally centrifuged at 3500 rpm for 1 h. The supernatant was then collected and freeze-dried to achieve a monolayer titanium carbide powder, denoted as T2D.

### 2.4 Preparation of Ti_3_C_2_T_*x*_ Quantum Dots (T0D)

The preparation of Ti_3_C_2_T_*x*_ quantum dots (T0D) followed a “top-down” solvothermal method.^41^ T2D (0.5 g) was added to a 250 mL three-neck flask containing 100 mL of deionized water. The mixture was stirred for 30 min to achieve a homogeneous dispersion. Air was removed by bubbling N_2_ for 1 h. Then, 3 mL of poly­(ether imide) (PEI) was added to the suspension and heated at 120 °C for 24 h via an oil bath with gentle stirring. A condenser tube was applied to reflux the evaporated liquid. After the system cooled, the obtained solution was vacuum-filtered with a 220 nm filter membrane, and the filtrate was further dialyzed with deionized water in a dialysis bag (retained molecular weight: 4000 Da) for 3 days to obtain a light yellow T0D aqueous solution. The T0D concentration was calculated by freeze-drying a certain volume of liquid and then adjusted with deionized water to 0.5 mg mL^–1^.

### 2.5 Preparation of T2D/CuO-# Composites and CuO

To prepare T2D/CuO-# composites, 0.04, 0.1, or 0.16 g of Cu­(NO_3_)_2_·3H_2_O was dissolved in 35 mL of 0.5 M NaOH and stirred for 30 min to obtain a copper precursor. T2D (0.1 g) was dispersed in 35 mL of deionized water by 30 min of stirring, and then, the above copper precursor solution was added into the T2D dispersion and stirred for 30 min to form a homogeneous solution. The mixture was then transferred to a Teflon-lined stainless-steel autoclave and heated at 200 °C for 20 h. Finally, the precipitate was washed three times with deionized water and dried at 70 °C overnight in a vacuum oven to obtain the T2D/CuO composite, denoted as T2D/CuO-1, T2D/CuO-2, or T2D/CuO-3, respectively. Pure CuO was also prepared without adding T2D. The nominal ratios of T2D/CuO-# composites are displayed in Table S1.

### 2.6 Preparation of T0D/CuO-# Composites

The preparation of T0D/CuO-# followed a straightforward self-assembly method. CuO was first prepared following the previous method; 0.1, 0.25, and 0.5 g of CuO were dispersed in 25 mL of deionized water by 30 min sonication and then mixed with 25 mL of 0.5 mg mL^–1^ T0D solution. After stirring for 24 h, the suspension was centrifuged at 10,000 rpm for 2 min. The sediment was then dried in a vacuum oven at 60 °C overnight to obtain T0D/CuO-1, T0D/CuO-2, and T0D/CuO-3, respectively. The nominal ratio (wt %) of T0D/CuO-# composites is displayed in Table S1.

### 2.7 Characterization

The crystalline phase of samples was examined by X-ray diffraction (XRD, Bruker D8 Advance), with Cu Kα radiation (λ = 1.54 Å). The surface chemical composition was characterized by X-ray photoelectron spectroscopy (XPS, AXIS SUPRA). The standard C 1s peak at 284.6 eV was used as the internal standard. The microstructures and morphologies of the samples were investigated by scanning electron microscopy (SEM, Hitachi S-4800) and transmission electron microscopy (TEM, FEI Tecnai G2 F20 S-TWINFEI). The elemental distribution analysis was performed by energy-dispersive X-ray (EDX) mapping. Atomic force microscopy (AFM, Agilent 5500AFM) was used to verify the thickness of monolayered nanosheets. Zeta potentials were tested by a Litesizer 500 particle analyzer. Brunauer–Emmett–Teller (BET) surface areas and pore size distributions were measured by nitrogen sorption analysis (ASAP2420 USA). UV–vis diffuse reflectance spectroscopy (DRS) of the samples was conducted using a Shimadzu UV-3000 spectrometer with an integrated sphere and barium sulfate (BaSO_4_) as a reference. The position of the valence band edge (*E*_*v*_) was measured by ambient photoemission spectroscopy (APS, KP Technology, SKP5050) using UV light irradiation (4.8–6.0 eV) and recording the photoemission signal. The cube root of the photoemission was extrapolated to zero in order to determine the value of *E*_*v*_. To measure the samples’ work function, contact potential difference measurements were carried out using a Kelvin probe, previously calibrated using a silver reference and APS.

Transient photocurrents and electrochemical impedance spectroscopy (EIS) were measured in three-electrode cells at an electrochemical workstation (CHI 760E) using an aqueous sodium sulfate solution (Na_2_SO_4_, 0.5 mol/L) as an electrolyte, a platinum (Pt) wire as a counter electrode, and a Ag^+^/AgCl electrode as a reference electrode. To prepare the working electrode, the samples were dispersed into suspensions containing 250 μL of ethanol, 250 μL of deionized water, and 50 μL of D-520 Nafion dispersion from DuPont. These dispersions were then evenly coated on 1 × 1 cm^2^ of indium-doped tin oxide-coated glass by drop casting and dried for 1 h at room temperature.

### 2.8 Photocatalysis

Photocatalytic hydrogen production experiments were carried out using a photocatalytic 160 mL glass reactor with a quartz window (Perfect Light Labsolar-6A) and a gas chromatograph with a thermal conductivity detector (GC9790II, PLF-01). For the reactions, 30 mg of the samples was added to a mixed solution containing 10 mL of triethanolamine (reagent grade) and 90 mL of deionized water. The system was evacuated first and purged with argon. A xenon lamp (300W, PLS-SXE300+) with a full spectrum (320 < λ < 780 nm) was used for 4 h of irradiation. For all experiments, a light source with a 16.60 cm^2^ illumination window was positioned 12 cm above the reactor to maintain a constant light intensity. For the recyclability experiment, after each reaction, samples were collected by centrifugation at 10,000 rpm for 5 min. After washing with 200 mL of deionized water twice, the residuals were dried in a vacuum oven at 60 °C for 12 h and reused for another round of testing. The symbol ± stands for the standard deviation.

## 3 Results and Discussion

The preparation of the photocatalytic materials under study is shown in Figure 1, outlining the successive steps to synthesize T2D, T0D, CuO, and their composites. The morphology of the prepared materials can be observed in Figure 2. SEM micrographs of T3D exhibit an "accordion" structure, as expected after the removal of aluminum layers from Ti_3_AlC_2_ (Figure 2a). TEM micrographs of T2D and T0D show 2D flakes of 1–3 μm and dots smaller than 10 nm accordingly (Figure 2b,c). SEM and TEM micrographs of T2D/CuO-2 exhibit a layering of 2–3 μm Ti_3_C_2_T_*x*_ nanosheets intermixed with smaller 500–800 nm CuO nanoflakes (Figure 2d–f). The CuO nanosheets are smooth and flat and lie on the surface of the T2D. The presence of 50–200 nm irregular particles may result from fragmentation during the hydrothermal method. TEM micrographs of T0D/CuO-2 show that T0D with an average size of 6.7 ± 1.9 nm is evenly distributed on the surface of CuO (Figure 2g–i). In comparison, T0D/CuO-2 shows a more regular morphology than T2D/CuO-2.

**Figure 1:**
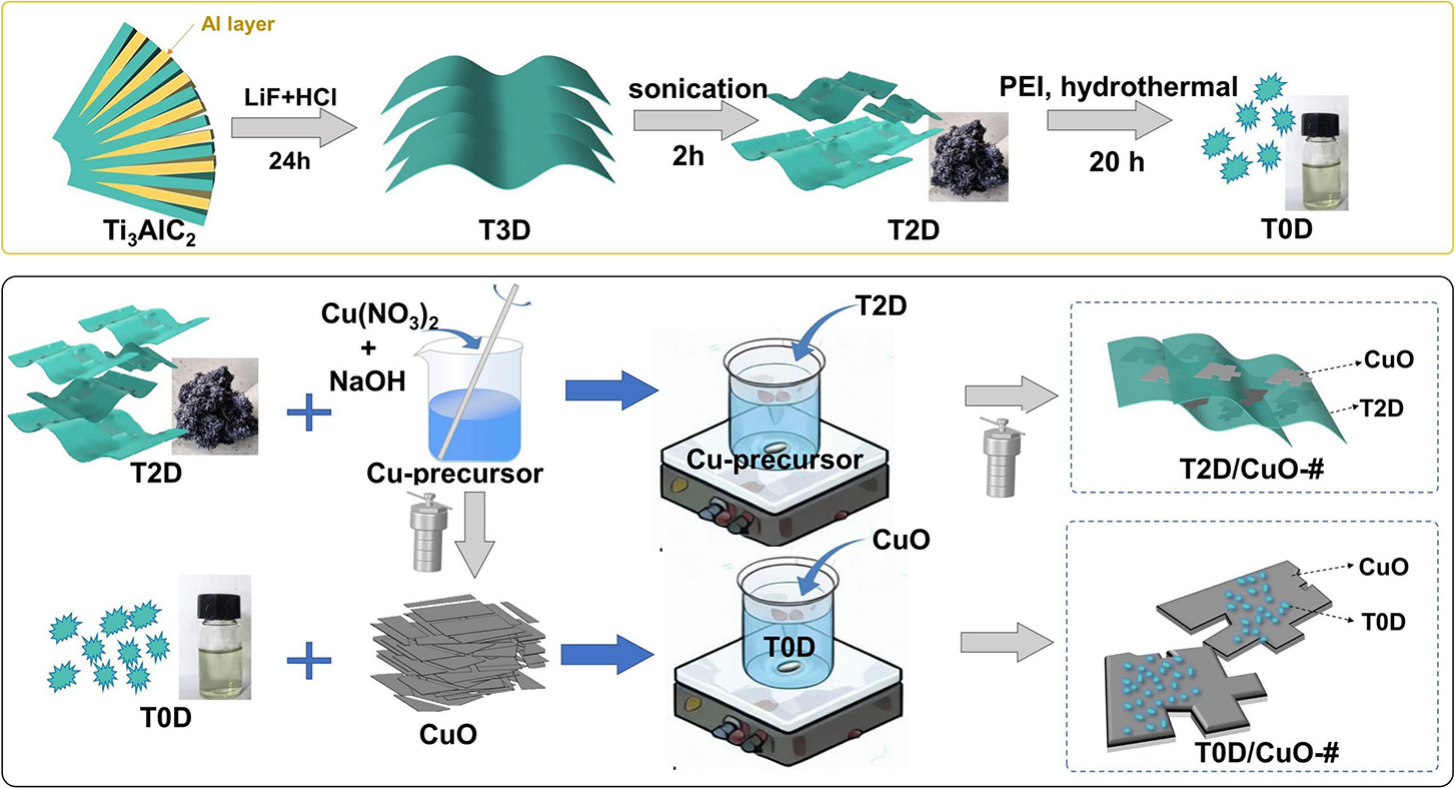
Preparation scheme of the T2D/CuO-# and T0D/CuO-# composites. PEI refers to poly­(ether imide). The multilayer Ti_3_C_2_T_*x*_, monolayer Ti_3_C_2_T_*x*_, and Ti_3_C_2_T_*x*_ quantum dots are denoted by T3D, T2D, and T0D, respectively.

**Figure 2:**
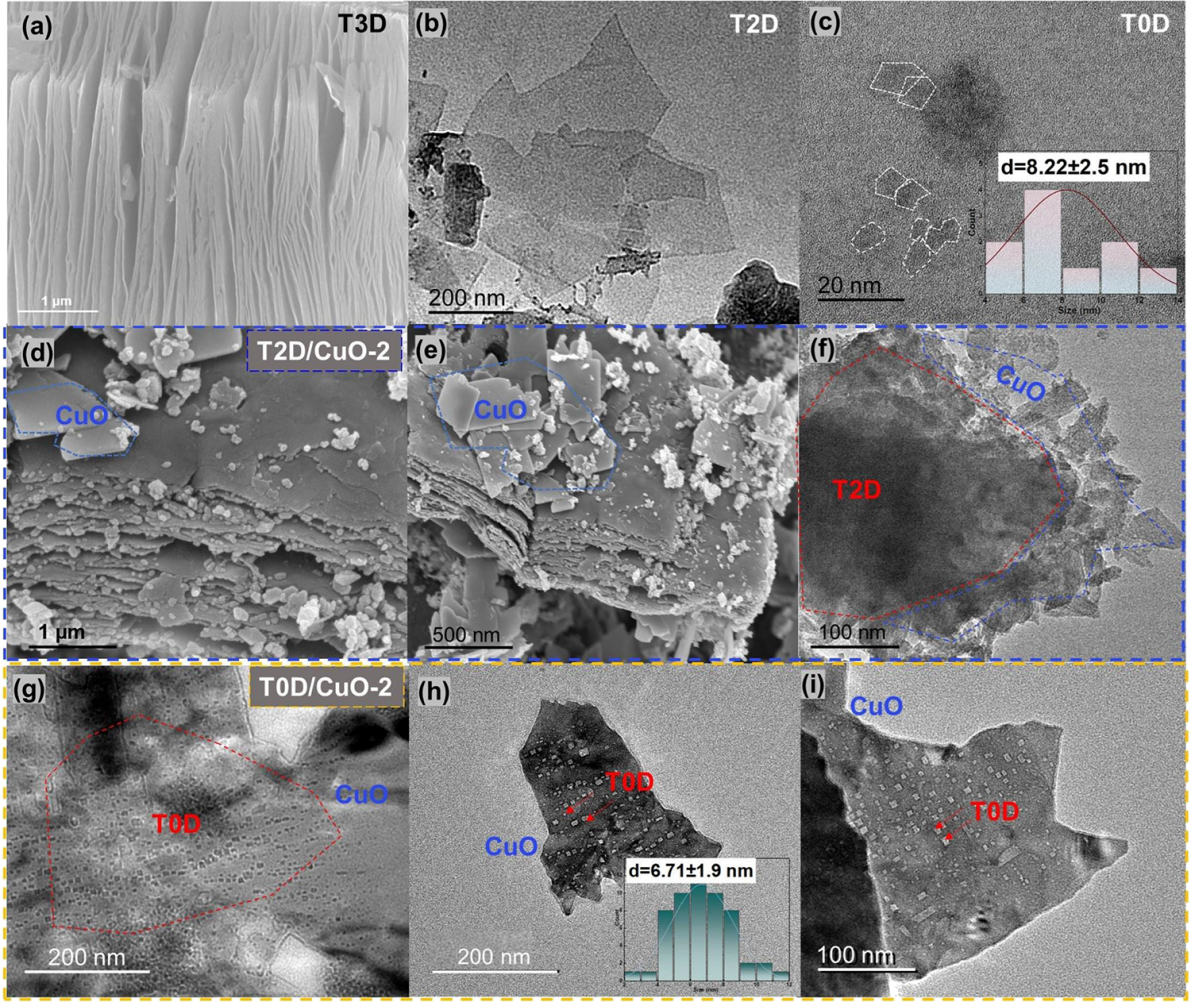
(a) SEM micrograph of T3D; (b) TEM micrographs of T2D and (c) T0D; (d, e) SEM and (f) TEM micrographs of T2D/CuO-2; (g–i) TEM micrographs of T0D/CuO-2.

Furthermore, TEM micrographs of T0D alone indicate a wide distribution of sizes centered at 8.2 ± 2.5 nm (Figure 2c). When composited with CuO in T0D/CuO-2, the size of T0D is similar, centered at 6.7 ± 1.9 nm (Figure 2g–i). The slightly smaller size may be attributed to the long stirring time during the composite preparation and the influence of CuO as a substrate aiding in better dispersion of T0D. Five mL of the T2D suspension was filtered with microporous water-based filter paper and air-dried naturally to obtain a self-supporting film with a radius of 2 cm as shown in Figure S1a,b. The surface has a metallic luster and a certain degree of flexibility. After centrifugation, the upper solution collected is dark green. After dilution, the mixture forms a light column after laser irradiation. The Tyndall effect and the self-supporting film indicate the successful preparation of T2D. The atomic force microscopy (AFM) micrograph shows that the thickness of T2D is 4.28 nm. The lattice spacing of 0.27 nm corresponds to the (0110) plane of Ti_3_C_2_T (Figure S1f).^37^ Micrographs of CuO and T0D/CuO-2 reveal no morphological changes at the SEM scale (Figure S1g,h), which is attributed to the small size of T0D. TEM micrographs of T0D/CuO-1 and T0D/CuO-3 reveal a noticeable difference in the loading amount of T0D on the surface (Figure S1i,j). EDX analysis of T0D/CuO-2 shows dots rich in Ti, confirming the successful addition of T0D onto CuO (Figure S2).

The FTIR spectra confirm that T2D and T0D, as well as CuO and T0D/CuO-2, exhibit similar internal chemical bonds and functional groups (Figure S3a,b). Next, the optical properties of T2D and T0D were investigated. The absorption of T2D and T0D is mainly in the ultraviolet region (Figure S3c), consistent with previous reports.^42–44^ The absorption of T0D is blueshifted compared with that of T2D, in agreement with its smaller dimensions and quantum size effects. Excitation-dependent fluorescence was measured on both samples: T2D had no fluorescence emission peak due to its metallic nature,^45^ whereas T0D exhibited excitation-dependent fluorescence behavior (Figure S3d). The emission peak of T0D gradually redshifts as the excitation wavelength increases from 350 to 490 nm. The strongest fluorescence peak appears at 475 nm when excited at around 370 nm. We assign the fluorescence emission of T0D to quantum confinement in their ultrasmall lateral dimensions or to surface defects.^46^ The fluorescence intensity depends on the band gap, which can be altered by changing the size of the QDs.^47^ Smaller QDs emit in the blue range, while larger dots emit in the red and near-infrared range.^48^ The T0D fluorescence results in Figure S3d suggest small-size QDs. Kim et al.^49^ concluded that the size of graphene QDs, exhibiting similar luminescence behaviors to MXene QDs, falls within the range of 5–10 nm when emitting blue fluorescence. This aligns well with the size distribution centered at 8.2 ± 2.5 nm obtained through TEM (Figure 2c). This correspondence confirms the synthesis of T0D, and the fluorescence behavior is attributed to quantum confinement effects.

The XRD patterns of the samples are shown in Figure 3a. The diffraction peaks at 9.5° (2θ) for both precursor Ti_3_AlC_2_ and T2D/CuO-2 align with the (002) crystal plane of Ti_3_AlC_2_ (ICSD no. 153266). The broadening of the peak and the decrease in intensity to disappearance in the composites can be attributed to the exfoliation and composite formation, which significantly reduces the stacking order along the *c*-axis and masks it from the XRD pattern. The XRD patterns of T0D/CuO-2 and CuO are highly consistent. The diffraction peaks at 35 and 38.2° correspond to the (002) and (111) crystal planes of CuO, respectively. Compared with the patterns reported in the literature (ICSD no. 67850),^50^ the peaks are shifted to a lower angle (2θ) by approximately 0.5°, indicating an increased lattice spacing. The XRD results of the other T0D/CuO-# and T2D/CuO-# samples are presented in Figure S4. The overlap of the characteristic peaks of the T0D/CuO-# composites indicates that they have a consistent crystal plane structure dominated by CuO. In the T2D/CuO-# composites, there is a distinct characteristic peak at 37.5°, coinciding with Ti_3_AlC_2_, indicating a higher crystalline content of Ti_3_C_2_T_*x*_ in T2D/CuO-# composites than in T0D/CuO-#.

**Figure 3:**
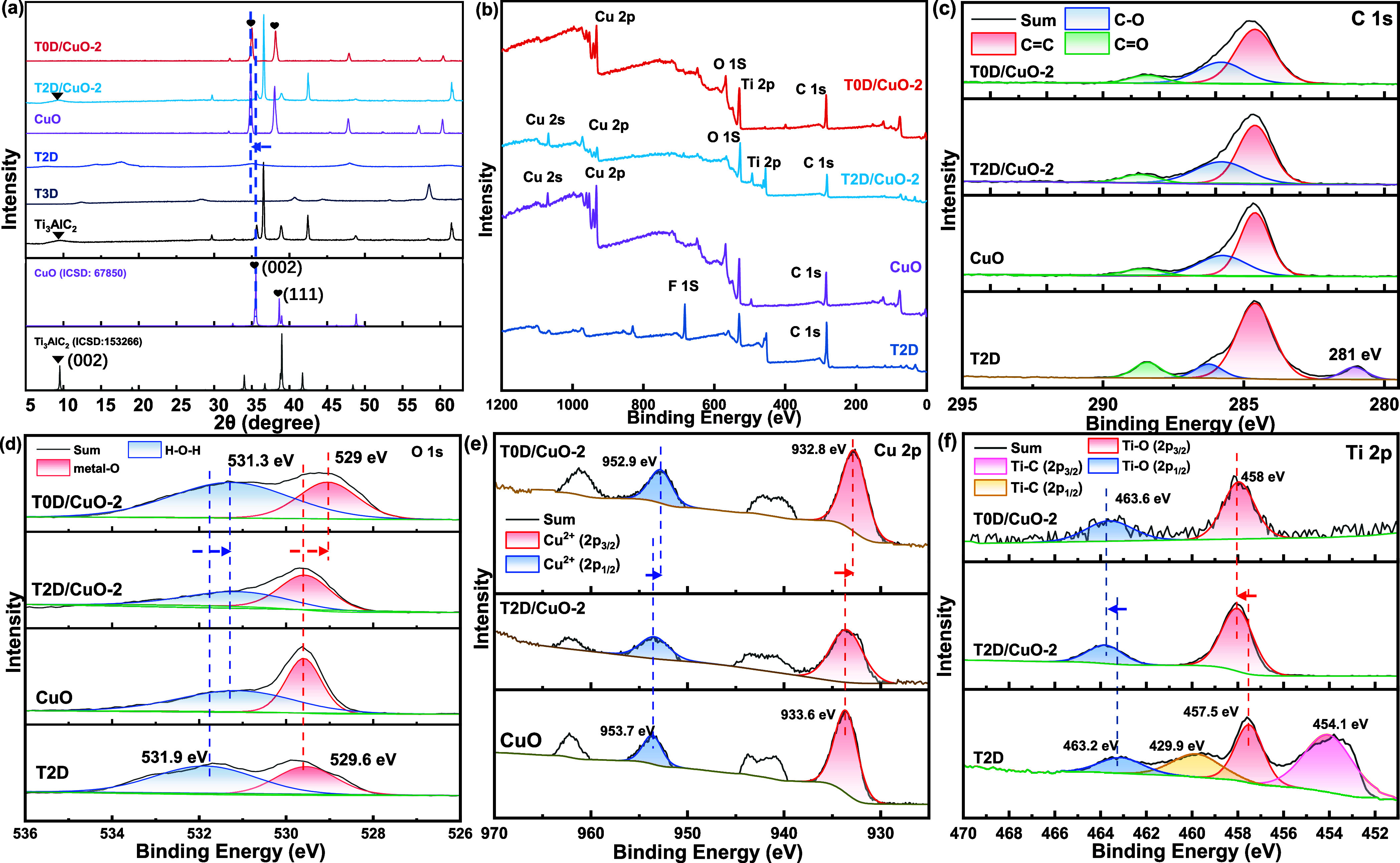
(a) XRD patterns of T2D/CuO-2, T0D/CuO-2, CuO, and Ti_3_AlC_2_; (b) XPS survey spectra of T2D/CuO-2, T0D/CuO-2, CuO, and T2D; high-resolution XPS spectra of (c) C 1s, (d) O 1s, (e) Cu 2p, and (f) Ti 2p on T2D/CuO-2, T0D/CuO-2, CuO, and T2D.

The chemical compositions and states of the samples are shown in Figure 3b–f. The C 1s spectrum of T3D reveals a C–Ti peak at 281.4 eV,^51^ which is absent in both T2D/CuO-2 and T0D/CuO-2 due to their higher level of oxidation (Figure 3c). The O 1s spectra of T2D/CuO-2, T0D/CuO-2, and CuO can be deconvoluted into two peaks, representing lattice oxygen in O-metal bonds and oxygen in hydroxyl groups (−OH).^52^ Compared with those of T2D, the peaks of T2D/CuO-2 and T0D/CuO-2 exhibit chemical shifts toward lower binding energies. The lattice oxygen peak in T0D/CuO-2 shifts from 529.6 to 529.1 eV, while the peaks of T2D/CuO-2 and CuO remain constant. In comparison to T2D/CuO, T0D/CuO-2 demonstrates a higher proportion of chemisorbed oxygen, indicating an increased OH content.^53^ The Cu 2p spectrum can be deconvoluted into two main peaks and two satellite peaks (Figure 3e). Peaks at 933.6 and 953.7 eV can be assigned to 2p_3/2_ and 2p_1/2_, respectively, in divalent copper. These peaks in T0D/CuO-2 are shifted to lower binding energy, reflecting an increased electron density on the CuO surface after forming a composite with T0D. Ti 2p spectra show that Ti–O peaks in T2D/CuO-2 and T0D/CuO-2, located at 457.4 and 463.5 eV, are shifted to higher binding energies compared with T2D, revealing a decrease in electron density.^45^ Ti–C peaks are prominent in T2D but are negligible in the composites. The XPS spectrum of T3D is displayed in Figure S5. T3D and T2D exhibit similar spectra. The presence of the F 1s peak indicates the residue F anion. Additionally, T3D shows C–Ti characteristic peaks similar to T2D (Figure S5b,c), which do not appear in T2D/CuO-2 and T0D/CuO-2, suggesting that the intensity of C–Ti chemical bonds decreases after compositing with CuO.

The results of the photocatalytic hydrogen production performance are illustrated in Figure 4. The T0D/CuO-# samples present the best performance with a hydrogen evolution that reaches an optimal 8696 μmol g^–1^ upon 4 h of irradiation on T0D/CuO-2. The T2D/CuO-# samples show inferior performance but still superior to CuO or T2D alone. The hydrogen production rate of the champion T0D/CuO-2 is 2174 (±189) μmol g^–1^ h^–1^, 24 times higher than CuO (91.3 μmol g^–1^ h^–1^) and 109 times higher than pure T2D (20 μmol g^–1^ h^–1^). This indicates that T0D can act as an efficient cocatalyst to improve the photocatalytic activity of CuO. Compared with T2D, T0D shows more promising effects in hydrogen production reactions. Recyclability tests were carried out to determine the stability of T0D/CuO-2 (Figure 4c). After each photocatalytic reaction, samples were collected by centrifugation, washed with distilled water, and dried before reusing. After five rounds of testing, T0D/CuO-2 still maintains a hydrogen evolution rate of 1134 μmol g^–1^ h^–1^, which is much higher than the initial rate of CuO alone (91.3 μmol g^–1^ h^–1^). SEM micrographs and XRD spectra before and after the photocatalytic reaction confirmed the morphology and structural stability of T0D/CuO-2 (Figure S6a,b). However, TEM micrographs clearly show a reduction in the amount of T0D distributed on CuO (Figure S6c,d), corresponding to a 47.8% decrease in the photoactivity. This reduction may be attributed to the loss of photocatalyst particles and/or the detachment of T0D during the centrifugation, washing, and drying processes. The comparison of the photocatalytic performance of similar systems, CuO-based photocatalysts and Ti_3_C_2_T_*x*_-based photocatalysts, is shown in Figure 4e. The results showed the excellent photocatalytic performance of CuO/Ti_3_C_2_T_*x*_ materials.^54–63^

**Figure 4:**
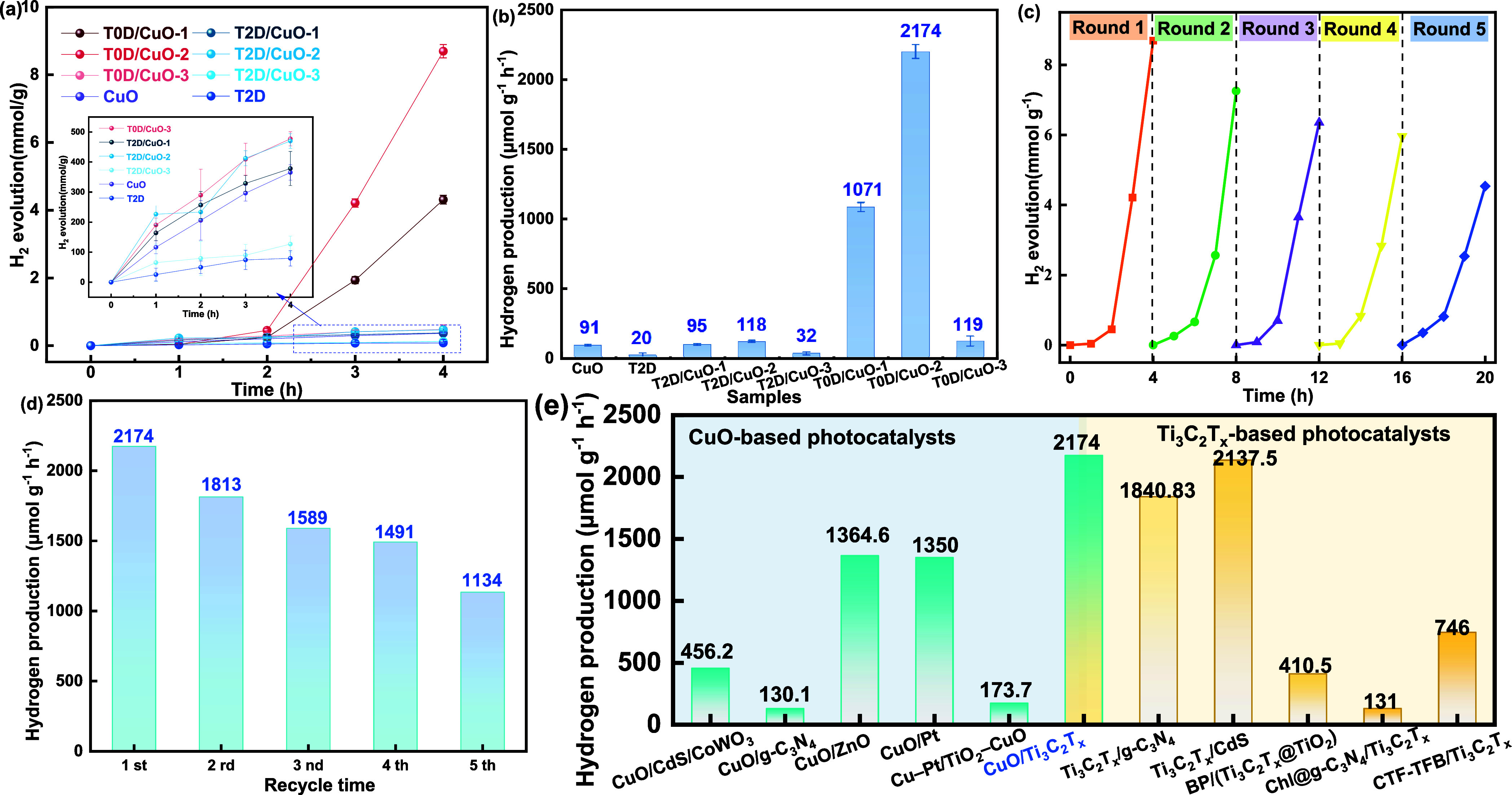
(a) Photocatalytic hydrogen production yield under full-spectrum illustration with an intensity of 100 mW cm^2^; (b) rates of CuO, T2D, T2D/CuO-#, and T0D/CuO-#; (c) recyclability test of T0D/CuO-2 photocatalytic H_2_ evolution in five cycles; (d) H_2_ production rate of each 4 h cycle at the same light irradiation intensity and sacrificial agent; (e) comparison of the photocatalytic performance of our work with similar systems (CuO-based photocatalysts and Ti_3_C_2_T_*x*_-based photocatalysts; ChI and CTF-TFB in the figure refer to chlorophyll and imine-linked covalent organic frameworks, respectively).

A control group was conducted to confirm that the superior performance of electrostatically prepared T0D/CuO-# compared with hydrothermally prepared T2D/CuO-# is not due to their different preparation methods or Ti_3_C_2_T_*x*_ MXene contents. T2D/CuO-2 composites were prepared electrostatically and hydrothermally using high (as in T2D/CuO-2) and low (as in T0D/CuO-2) Ti_3_C_2_T_*x*_ MXene contents. The T2D/CuO-2 composites electrostatically prepared exhibit a lower hydrogen production rate than the ones hydrothermally prepared (Figure S7a). Moreover, the T2D/CuO-2 composites with a lower Ti_3_C_2_T_*x*_ MXene content produce less hydrogen. Therefore, the superior properties of T0D/CuO-# samples compared with T2D/CuO-# samples in Figure 4 are attributed to the physical properties of its components and not to their preparation method or Ti_3_C_2_T_*x*_ MXene content. We also measured the zeta potential of different materials at pH 7. CuO is positively charged, whereas T3D, T2D, and T0D are negatively charged, indicating that electrostatic bonding is possible (Figure S7b).

The surface areas of the materials were characterized by a nitrogen sorption analysis (Figure 5a). Most adsorption and desorption occur at high pressures above 0.6 *P*/*P*_0_ assigned to nitrogen condensation on the surfaces of the materials. T2D/CuO-2 exhibits stronger hysteresis, assigned to its layered aggregated structure with interlayer pores, as observed by SEM (Figure 2). For further insights, the N_2_ sorption curves of other T0D/CuO-# and T2D/CuO-# materials are provided in Figure S8. The surface areas of T0D/CuO-1, T0D/CuO-2, and T0D/CuO-3 are 43.2, 34.5, and 12.0 m^2^ g^–1^, respectively, and that of CuO is 7.0 m^2^ g^–1^. Therefore, T0D/CuO-2 has a 5-fold increase in the surface area compared to CuO, resulting in more active sites. Compared with T2D/CuO-#, T0D/CuO-# samples have larger surface areas, thus confirming that smaller cocatalyst sizes result in larger composite surface areas.

**Figure 5:**
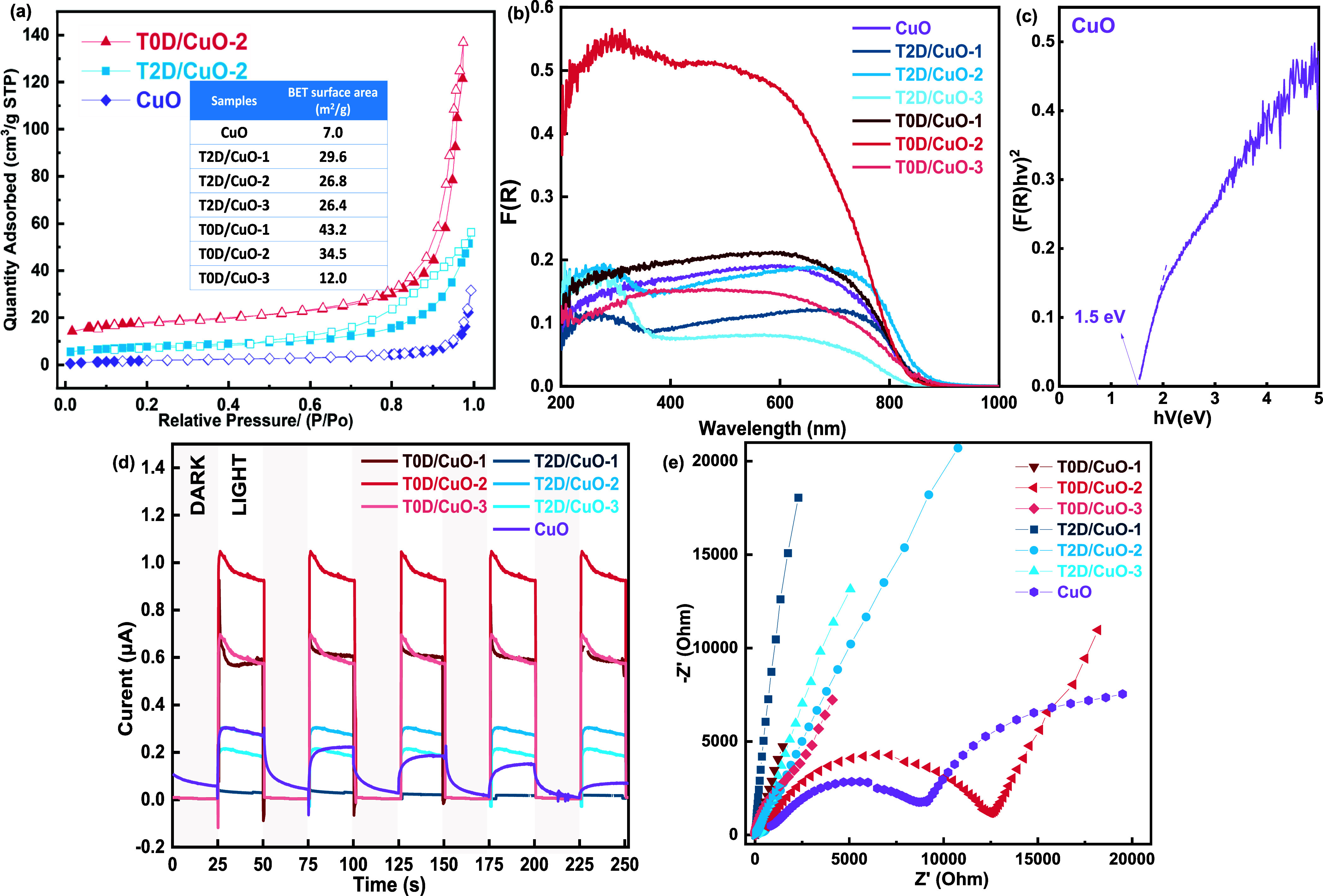
(a) N_2_ sorption isotherms of CuO, T2D/CuO-2, and T0D/CuO-2. The inset includes BET surface areas of all samples (solid and open symbols represent adsorption and desorption branches, respectively); (b) UV–vis reflection spectra of T2D/CuO-#, T0D/CuO-#, CuO, and T3D; (c) Tauc plot of CuO; (d) transient photocurrent response curves at 0 V vs the Ag/AgCl reference electrode; (e) Nyquist plots of T2D/CuO-#, T0D/CuO-#, and CuO (the electrolyte: 0.5 M Na_2_SO_4_ with the pH of 7).

Ultraviolet–visible (UV–vis) spectroscopy in diffuse reflectance mode was performed to explore the optical properties of the photocatalysts. The Kubelka–Munk function *F*(*R*), analogous to absorption, was calculated and is plotted in Figure 5b. T0D/CuO-2 has better absorption capabilities in both the ultraviolet- and visible-light regions (<800 nm) compared to pure CuO, indicating that the T0D loading promotes the light absorption in the composite, in agreement with their highest photocatalytic response (Figure 4). Pure CuO has a wide absorption range from 200 to 800 nm, and a similar trend can be seen in the composites. T2D/CuO-# shows an adjacent absorption edge at around 360 nm, maybe due to parasitic light absorption by T2D. The light absorption capacity of T2D/CuO-# is primarily determined by T2D due to its high content (Table S1). Conversely, the absorbance of T0D/CuO-# is mainly attributed to CuO. The absorption of T0D/CuO-3 is lower than CuO, indicating that excessive cocatalyst loading can reduce the light absorption performance of the composite. The CuO band gap was estimated through linear extrapolation to zero on Tauc plots of [*F*(*R*)·*h*υ]^1/*n*^ against photon energy, using *n* = 1/2 for direct transition (Figure 5c).^64^ The band gap of CuO was determined to be 1.5 eV, in agreement with the literature.^65,66^

Transient photocurrent response curves and impedance spectra of the photocatalysts were measured on prepared films on a conductive substrate. T2D/CuO-2 shows the most prominent photocurrent response (Figure 5d), in agreement with its superior photocatalytic response and light absorption (Figures 4 and 5b). The photocurrent intensities of T0D/CuO-# are substantially larger than those of T2D/CuO-#, showing a higher charge separation efficiency. The Nyquist plots show that the semicircle radius of T0D/CuO-2 is significantly smaller than that of other composites and the semicircle radius of T2D/CuO-2 is the smallest among the T2D/CuO-# materials. T0D/CuO-2, therefore, has lower charge transfer resistance and higher conductivity than other composites (Figure 5e).

We applied ambient photoemission spectroscopy (APS) and Kelvin probe measurements to determine the valence band edge (*E*_*v*_) and work function (*E*_*f*_) of the composite components (Figure 6a,b). The *E*_*v*_ of CuO is at −5.76 eV vs vacuum, and its work function is 5.24 eV. Combined with the band gap value, the energy band diagram of the composite photocatalyst was constructed (Figure 6c). The measured values are listed in Table S2. Due to their metal-like property, T2D and T0D were only characterized by a Kelvin probe,^67^ showing work functions of 4.66 and 3.94 eV, respectively. The Fermi levels of CuO and T2D or T0D should equilibrate upon contact, as represented in Figure 6c. According to the deeper Fermi level of CuO compared to those of T2D or T0D, this equilibration would lead to a downward band bending (toward the interface) in the CuO. This is also supported by the Cu 2p XPS peak shift (Figure 3e). This downward band bending means that an interfacial electric field is available to enhance the charge separation and transfer of photogenerated electrons from CuO to Ti_3_C_2_T_*x*_ T2D or T0D upon irradiation. The enhanced charge separation can reduce e^–^/h^+^ recombination in CuO, thus improving the photocatalytic activity of the composite material. T0D possesses the lowest work function compared to T2D, indicating the potential for larger band bending within the CuO/T0D interfacial electric field compared to the CuO/T2D composites. The larger band bending suggests enhanced electron transfer and reduced recombination, providing additional evidence for T0D/CuO exhibiting the highest photocatalytic activity (Figure 4) and photocurrent (Figure 5d).

**Figure 6:**
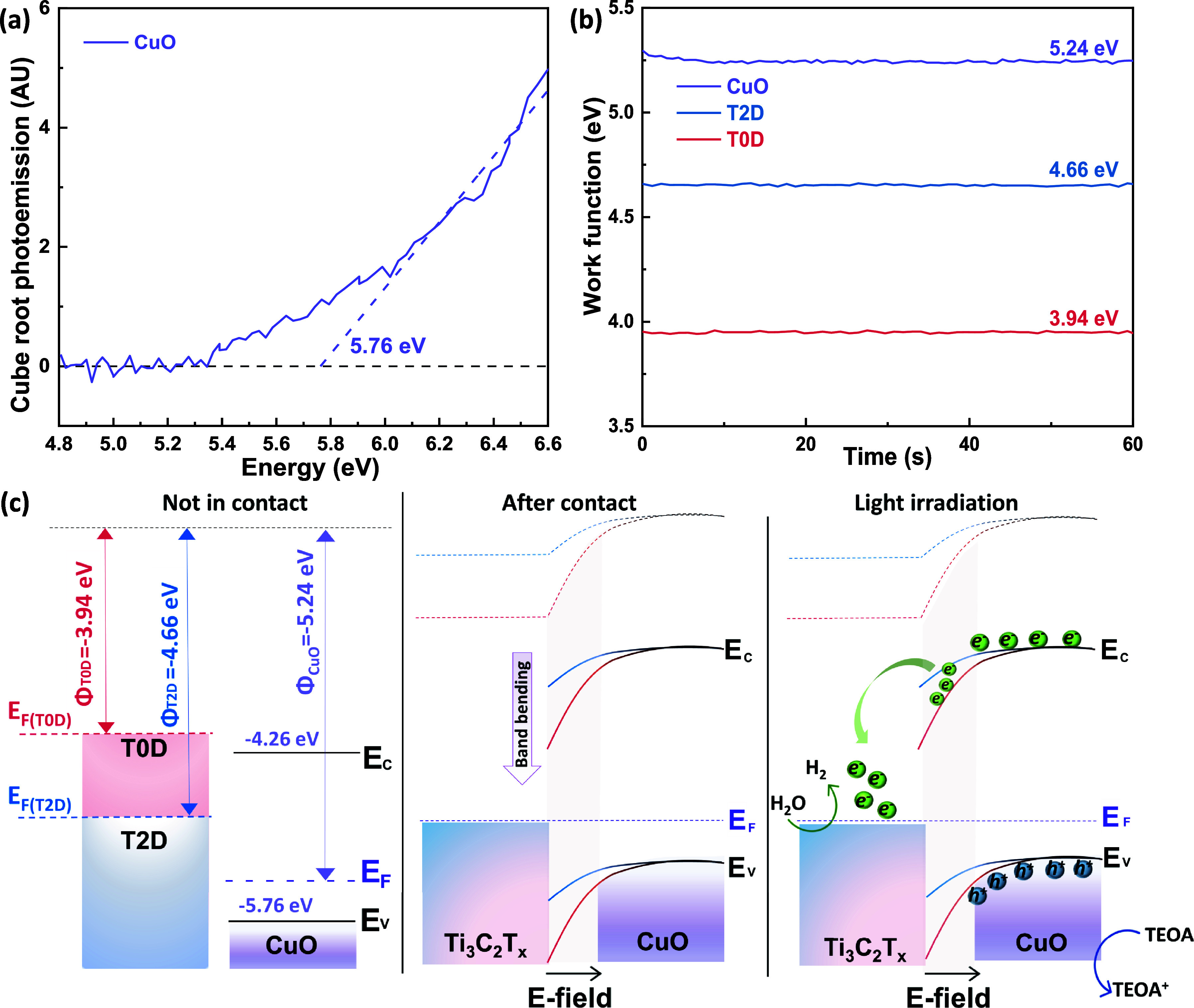
(a) Ambient photoemission spectrum of CuO; (b) work functions of CuO, T2D, and T0D; (c) energy band diagram of Ti_3_C_2_T_*x*_ in different dimensions and CuO before and after contact with Fermi level equilibration and light irradiation.

In summary, we can attribute the enhancement in photocatalytic activity of T0D/CuO-# to a preferential flow of electrons, efficient e^–^/h^+^ separation, and sufficient contact area. The built-in electric field established at the T0D/CuO interface enhances electron flow and mitigates the rapid recombination within CuO. Simultaneously, the small size of T0D ensures a broad contact area, more active sites, and reduced parasitic absorption capability of T0D/CuO, culminating in higher overall photocatalytic activity. T2D and T0D exhibit lower work functions compared to CuO, suggesting that the morphology does not alter the reaction mechanism of Ti_3_C_2_T_*x*_ as a cocatalyst in photocatalytic reactions. However, the lowest work function of T0D renders it more suitable as an electron acceptor than the multidimensional Ti_3_C_2_T_*x*_, facilitating faster electron transfer and thereby prolonging the lifetime of photogenerated electrons in CuO.

## 4 Conclusions

In this study, we successfully demonstrated the synthesis of Ti_3_C_2_T_*x*_ nanosheets/CuO (T2D/CuO) and Ti_3_C_2_T_*x*_ quantum dots/CuO (T0D/CuO) photocatalytic composites, in which CuO works as a light absorber and Ti_3_C_2_T_*x*_ as a cocatalyst. By preparing different Ti_3_C_2_T_*x*_ dimensionalities (T2D and T0D) and compositing them with CuO, we found that when Ti_3_C_2_T_*x*_ nanosheets are broken down to a quantum dot size, they can be tightly loaded on CuO through a simple electrostatic binding method. At the same time, UV–vis spectroscopy and BET results confirm that T0D does not affect the light absorption of CuO but provides large surface areas. The optimal T0D/CuO sample achieved a hydrogen production rate of 2174 (±189) μmol g^–1^ h^–1^, which is 19 times higher compared to the optimal T2D/CuO sample and more than 108 times higher than that of pure CuO. The enhanced performance is attributed to the increased active sites, the efficient light absorption, and the enhanced charge separation. Due to the suitable Fermi levels, the photogenerated electrons on the conduction band of CuO are quickly transferred to T0D, thereby obtaining an effective charge carrier separation. The electrons generated by CuO are utilized for the reduction of water to produce hydrogen in the presence of hole scavengers. This work highlights the impact of Ti_3_C_2_T_*x*_ dimensionalities on its cocatalytic performance and demonstrates why quantum dot-sized Ti_3_C_2_T_*x*_ is a better cocatalyst for photocatalytic hydrogen production.

## Supplementary Material



## Notes

Comparative elemental composition profiles of T2D/CuO-# and T0D/CuO-# composites with structural validation via AFM and TEM, photocatalytic hydrogen production rates correlated with staggered band alignment and interfacial charge transfer, and UV–vis absorption thresholds and excitation-dependent emission confirming quantum confinement effects in T0Ds (PDF)
