# Supplementary material for: Ti3C2T x 2D and 0D MXene Cocatalysts on CuO for Enhanced Photocatalytic Hydrogen Evolution
Source: Energy Fuels. 2025 Jun 6;39(24):11855–64. doi: 10.1021/acs.energyfuels.5c01244 (PMC12186294; doi:10.1021/acs.energyfuels.5c01244)
Supplement: Supplementary file 1 [file ef5c01244_si_001.pdf]

Supporting Information

$Ti_3C_2T_x$ 2D and 0D MXene co-catalysts on CuO for enhanced photocatalytic hydrogen evolution

Lu Chen[1,2], Taotao Qiang[1*], Matyas Daboczi[2], Yasmine Baghdadi[2], Salvador Eslava[2*]

1 Institute of Biomass & Functional Materials, Shaanxi University of Science & Technology, Xi'an, Shaanxi, 710021, PR China

2 Department of Chemical Engineering, Imperial College London, London SW7 2AZ, United Kingdom

*Corresponding author

Email: qiangtt515@163.com; s.eslava@imperial.ac.uk

Table S1. Nominal wt% elemental content in T2D/CuO-# and T0D/CuO-# composites preparation

	**Nominal Content *(wt%)***			
**Materials**	Ti	Cu	O	Other (C, F, Cl...)
T2D/CuO-1	75.7	9.3	2.3	12.7
T2D/CuO-2	64.4	19.8	5	10.8
T2D/CuO-3	56.1	27.6	6.9	9.4
T0D/CuO-1	9.5	71	17.9	1.6
T0D/CuO-2	4.1	76.2	19	0.7
T0D/CuO-3	2.1	78	19.6	0.3

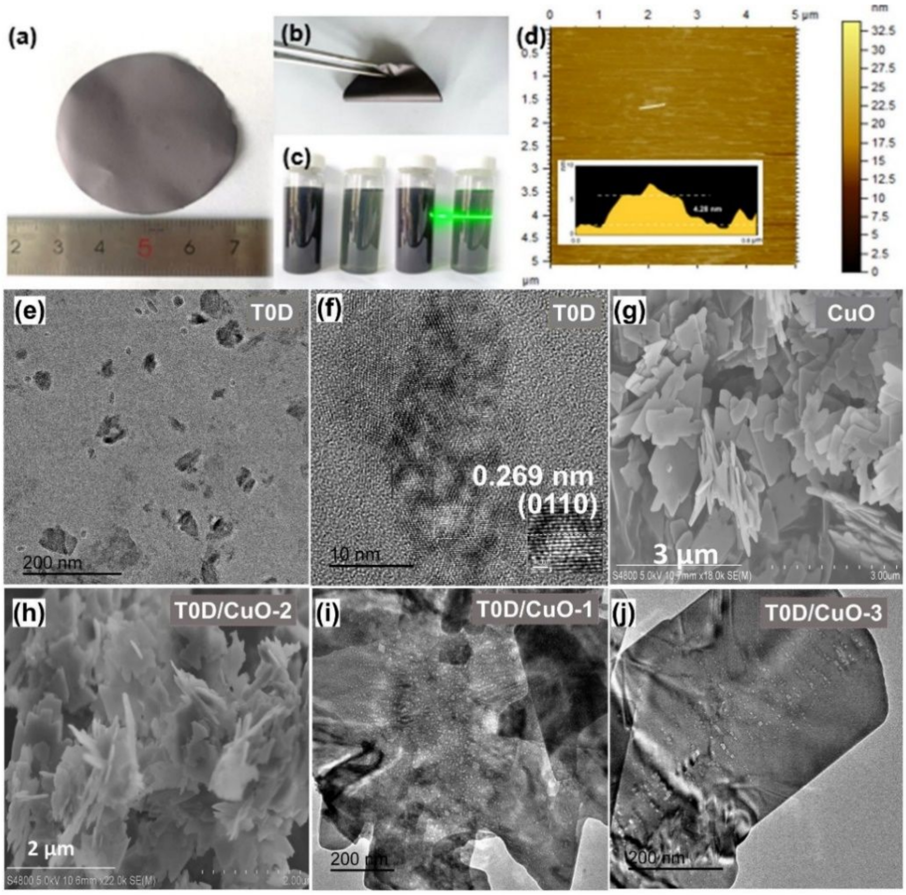

Fig. S1 (a, b) The self-supporting film of T2D; (c) the T2D solution and its Tyndall effect; (d) AFM micrograph of T2D; (e) TEM and (f) HRTEM micrographs of T0D, and particle size distribution in the inset in (f); (g) SEM micrograph of CuO nanosheets; (h) SEM micrograph of T0D/CuO-2; (i, j) TEM micrographs of T0D/CuO-1 and T0D/CuO-3.

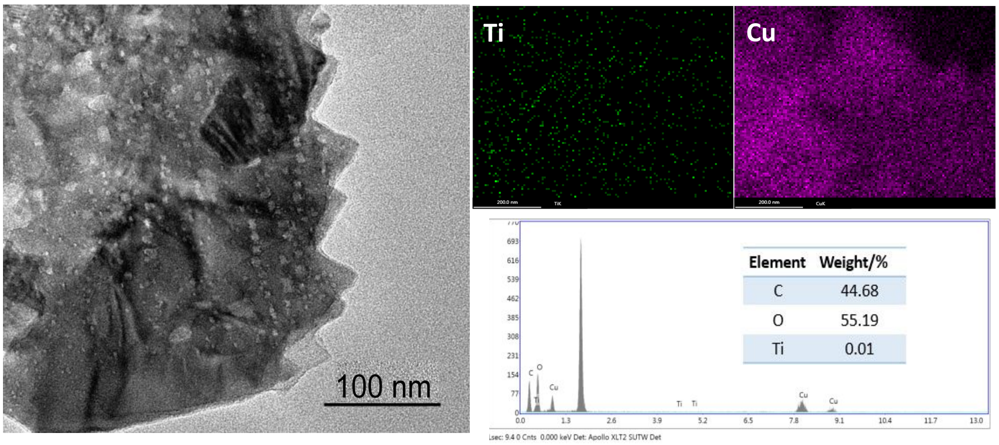

Fig. S2 SEM and EDX micrographs of T0D/CuO-2.

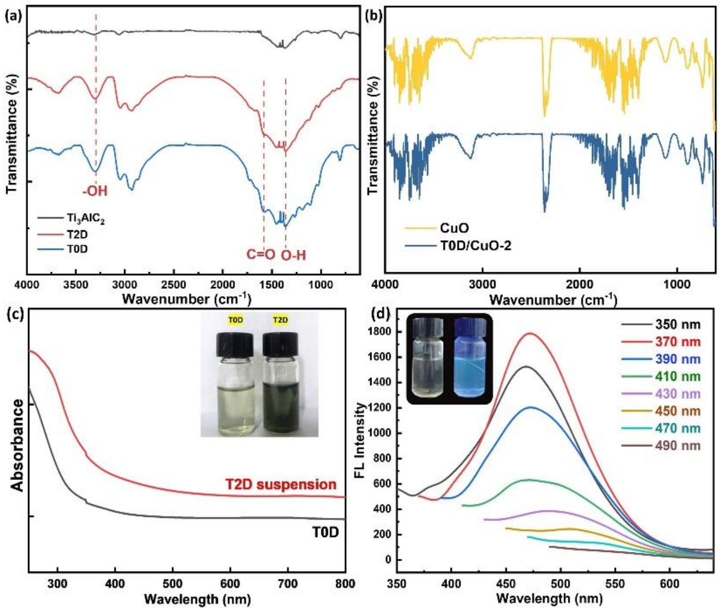

Fig. S3 (a) UV-vis spectra of T2D and T0Ds solution and (b) excitation-dependent emission spectrum of T0Ds solution.

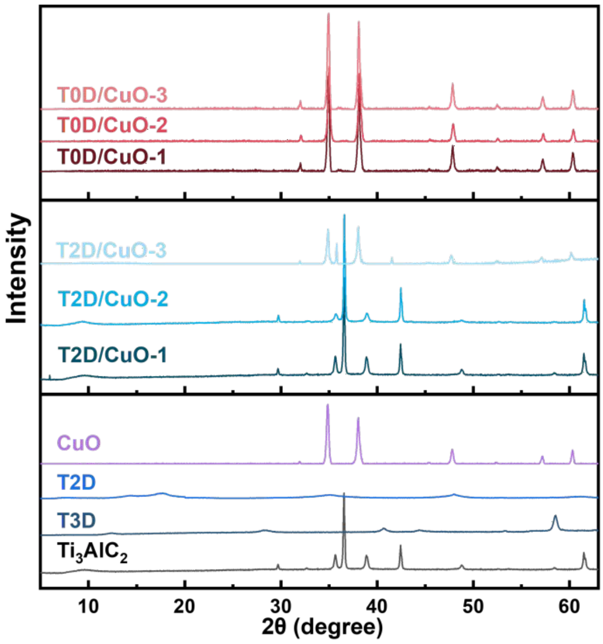

Fig. S4 XRD results of T2D/CuO-1, T2D/CuO-3, and T0D/CuO-1, T0D/CuO-3, CuO and Ti_3AlC_2

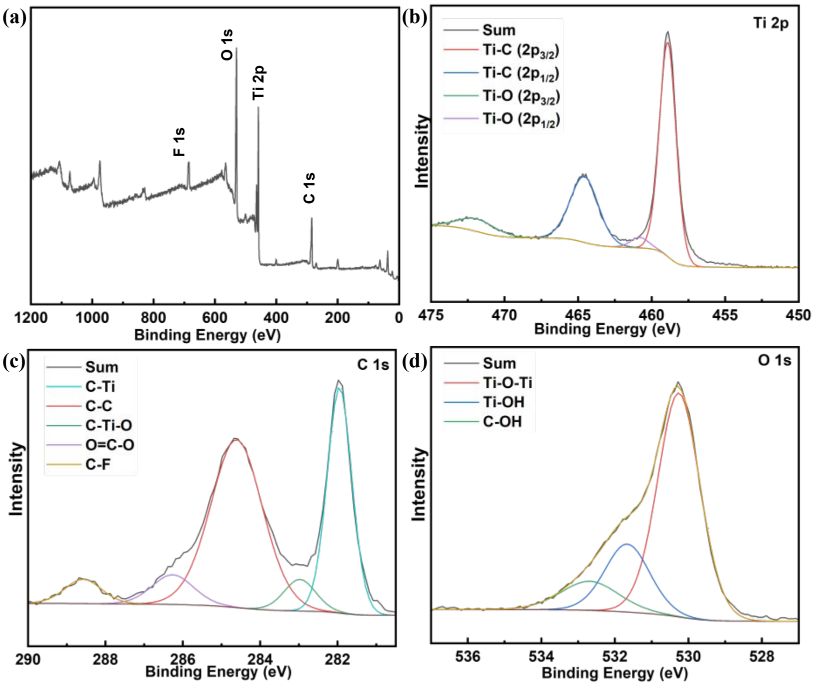

Fig. S5 (a) XPS survey and (b-d) high-resolution Ti 2p, C 1s, and O 1s spectra of T3D.

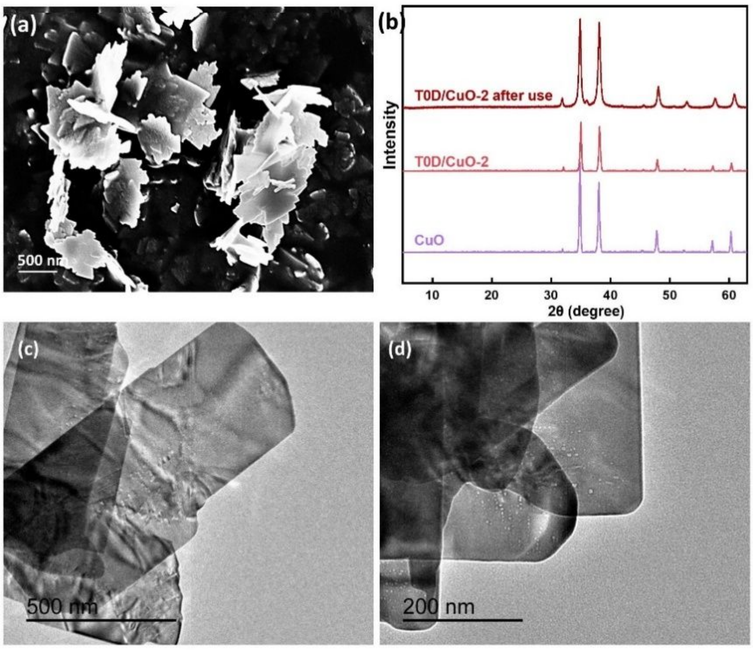

Fig. S6 (a) SEM micrograph and (b) XRD patterns of T0D/CuO-2 after photocatalytic reaction. Note: shift in "after use" pattern is attributed to sample height misalignment.

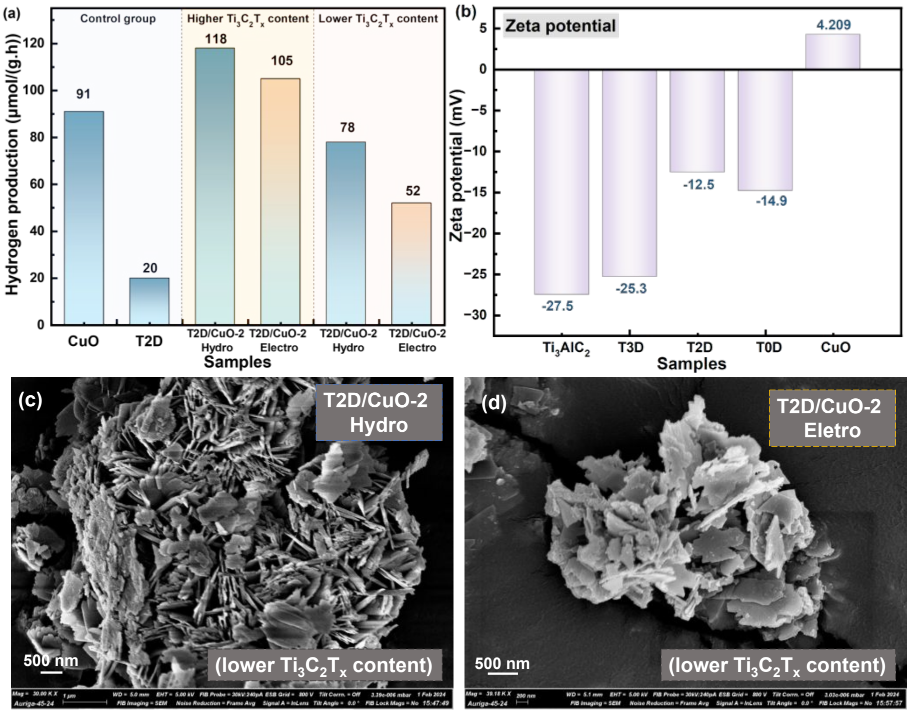

Fig. S7 (a) Photocatalytic hydrogen production rate of CuO, T2D and T2D/CuO-2 prepared through hydrothermal and electrostatic bonding methods with different $Ti_3C_2T_x$ content under full-spectrum illustration with the intensity of 100 mW cm^2, (b) Zeta potential plots of Ti_3AlC_2, T3D, T2D, T0D and CuO in aqueous dispersions (pH=7, 0.01 mg/mL); SEM micrographs of T2D/CuO-2 with lower $Ti_3C_2T_x$ content prepared through hydrothermal and electrostatic bonding methods.

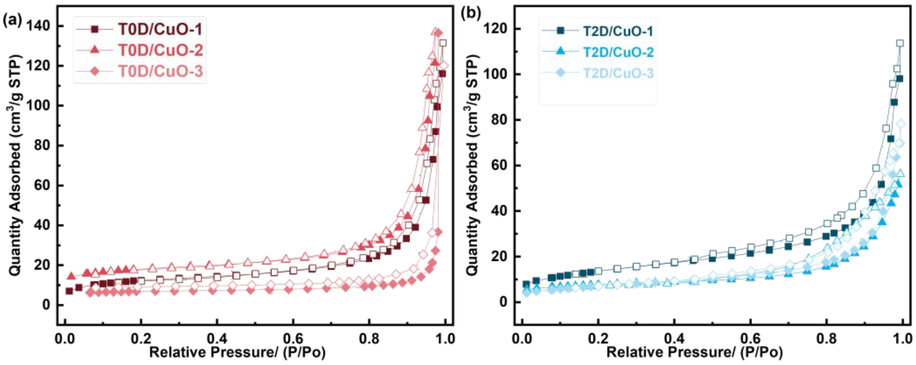

Fig. S8 N_2 sorption isotherms of T2D/CuO-# and T0D/CuO-# (solid and open symbols represent adsorption and desorption branches, respectively).

Table S2 Energy band positions and energy band gaps of CuO and T0D

Materials	**CuO**		**T0D**	
E (eV)	vs. Vacuum	vs. NHE (pH=7)	vs. Vacuum	vs. NHE (pH=7)
Fermi level	-5.24	0.8	-3.96	-0.48
Eg	1.5	/	/	/
VB	-5.76	1.32	/	/
CB	-4.26	-1.16	/	/
